# Clinical Assessment of Dental Implant Stability During Follow-Up: What Is Actually Measured, and Perspectives

**DOI:** 10.3390/bios8030068

**Published:** 2018-07-13

**Authors:** Elisabetta M. Zanetti, Giulia Pascoletti, Michele Calì, Cristina Bignardi, Giordano Franceschini

**Affiliations:** 1 Dipartimento di Ingegneria, Università di Perugia, 06125, Italy; giulia.pascoletti@studenti.unipg.it (G.P.); giordano.franceschini@unipg.it (G.F.); 2 DIEEI, Università di Catania, 95125, Italy; mcali@diim.unict.it; 3 DIMEAS, Politecnico di Torino, 10129, Italy; cristina.bignardi@polito.it

**Keywords:** implant stability, osseointegration, modal analysis, resonance frequency, ultrasound, reverse torque, functional loading, early loading, damping

## Abstract

The optimization of loading protocols following dental implant insertion requires setting up patient-specific protocols, customized according to the actual implant osseointegration, measured through quantitative, objective methods. Various devices for the assessment of implant stability as an indirect measure of implant osseointegration have been developed. They are analyzed here, introducing the respective physical models, outlining major advantages and critical aspects, and reporting their clinical performance. A careful discussion of underlying hypotheses is finally reported, as is a suggestion for further development of instrumentation and signal analysis.

## 1. Introduction

The success or failure of bone implants has been demonstrated to be related to the quality of the bone–implant interface which provides the support to transfer loads from the implant to the bone [1]. New bone apposition at the bone–implant interface requires a good primary implant stability with limited micromovements at the interface; this primary stability is provided by the mechanical engagement of the implant in the bone [2]. In facts, relative displacements between the bone and the implant above 50–150 µm [3] can lead to fibrous bone formation, providing a very poor long-term secondary stability [4]; secondary stability is the biologic stability provided through bone regeneration and remodeling. The necessity of limiting these so-called ‘micromovements’ has induced the setup of follow-up protocols where functional loads are applied after a prescribed period of time (3–6 months, according to the original protocol described in [5]).

On the other hand, loads have been proven to provide the necessary stimulus for bone maturation and for bone remodeling [6]; therefore, too-long ‘resting’ times could lead to longer healing times, and could even potentially induce bone resorption [7,8].

Various implant surface treatments have been introduced, based on both additive and subtractive technologies [9], mainly following three aims. The first aim is increasing surface roughness in order to improve bone anchoring to the implant (see, for example, grit-blasting and acid-etching [10] and *3D* printing techniques [11,12]); the second aim is to promote bone in-growth through bone-mimicking surfaces as realized by layer deposition through titanium plasma-spraying, anodization, or calcium phosphate coatings [10]; the final aim is surface functionalization that enables the controlled release of drugs in the peri-implant area. All these treatments, tested in the clinical practice, have been proven to be able to reduce osseointegration times [10,13]. In the meantime, also the impact of surgical technique is being clarified, leading to the optimization of drilling speed [14] and implant site size [15]. All these improvements have finally lead to the ‘early loading’ concept, or even to ‘immediate loading’ [10,16,17]. New loading protocols are therefore being established for patient follow-ups, in relation to the specific implant surface.

Finally, it should be added that the bone response is patient-specific, since it depends on his/her age and health [18] and on details of the respective surgery [19,20,21]. Therefore, patient-specific follow-ups should be designed.

All the above reported considerations lead to the conclusion that the time required for implant osseointegration might be quite variable and that setting up an experimental method for a quantitative assessment of implant stability would be recommendable. This approach would imply a double advantage: on one hand, the risk of loading the implant too early would be avoided; on the other hand, time before loading could be reduced with minor discomfort for the patient and faster healing times. Finally, setting up an experimental method to test implant stability would also allow continuous monitoring of implants and early interventions in case degenerative phenomena are taking place.

In recent times, various devices for the quantitative assessment of osseointegration during follow-up have been developed; the aim of this review is clarifying the physical principle behind each technique and discussing their use in detail, along with the respective validation trying to establish limits and advantages for each device and the reliability of the respective measurements.

## 2. Methods

The gold standard to assess osseointegration is the histologic analysis [22], since the term ‘osseointegration’ itself has been defined in relation to images observable by a light microscope. On the other side, this is not a viable technique in the clinical practice, with reference to human subjects, where radiographic examination is likely to be the most common diagnostic tool [23,24].

Theoretically, there is no alternative way to ascertain the type of contact between bone and the implants. However, the invasiveness of these methods and their inherent limits (for example, diffraction phenomena occurring at the bone–implant interface due to the presence of metal) have driven the focus of attention towards the experimental measurement of implant stability, since mobility is the most evident clinical sign allowing an indirect assessment of osseointegration. Methods used to assess bone quality prior to implantation are not discussed here, since implant follow-up is mainly examined.

### 2.1. Methods for the Direct Assessment of Osseointegration

#### 2.1.1. Histologic Analysis

Histology is a very invasive method, since it would require making a biopsy. For this reason, its current use has been limited to experimental and nonclinical studies, even if it allows determining osseointegration level with high accuracy. Therefore, it is not going to be analyzed here differently from the clinically applicable methods.

#### 2.1.2. X-Rays

Among the various diagnostic exams based on X-rays, according to [25,26], the most common techniques are listed here as follows:

■ Periapical radiography
■ Panoramic radiography
■ Occlusal radiography
■ Cephalometric radiography
■ Conventional tomographic radiography
■ Computed tomography (*CT*)
  - Medical *CT*
  - Cone-beam computed tomography (*CBCT*)
■ Magnetic resonance imaging (*MRI*)

Some common issues can be outlined: first of all, standard radiographs are not calibrated, and there is no reference phantom so that absolute bone density values cannot be indicated; in addition, metal parts produce a significant noise in the immediate proximity of the implant.

Dealing more specifically with the first four methods, they are classified as “planar imaging modalities”, being able to produce a two-dimensional projection of the system anatomy. The output of radiographs is therefore related to bone density distribution, but being a projective view, the information given by these exams is actually a ‘sum’ (or integral) of tissue density in the projection direction [27]. Some uncertainties influence these *2D* techniques: film placement errors (periapical and occlusal techniques), patient positioning errors (panoramic radiography), and influence of the operator ability (cephalometric technique). Moreover, even if high-resolution images are generated, they still are affected by distortion and magnification errors, and in most cases, they allow one to investigate only a limited portion of the jaw. For example, the periapical radiography produces a lateral view of the alveolus, but it is not able to give cross-sectional data, whereas the occlusal technique generates an inherently oblique and thus distorted maxillary occlusal view. As a result of the outlined limitations, it is commonly assumed that X-rays can only detect variation of bone density in excess of 40% [24]. Nonetheless, radiographs can produce some distinctive features with a specific clinical value: well-osseointegrated implants appear radiopaque, with no radiolucency lines between the implant and bone, since these are an index of implant mobilization [23]; the bone proximity to the implant can be assessed as well as the homogeneity of its trabeculation. Additionally, crestal bone height is an important predictor of implant success [28]. However, as stated above, the buccal surface cannot be examined, and this surface is of utmost importance for implant stability [24]. Secondly, crestal bone height is expected to change 0.1 mm per year after the first post-implant year, and common radiographs do not reach this accuracy, due to the above cited optical distortions [29].

On the other side, *3D* methods are classified as quantitative accurate techniques, producing exact and non-distorted tomographic sections. The *CT* technique produces tomographic sections that allow one to differentiate and quantify hard and soft tissues. This technique has been developed over time also for dental analysis applications, moving from the medical *CT* to the *CBCT*. In fact, medical *CT* imaging requires a considerable radiation exposure for the patient, while *CBCT* is less invasive; it consists of a cone-shaped X-ray beam which rotates 360° around the patient. This method allows one to limit the average absorbed radiation dose with respect to other techniques (the radiation is about 25% of that of a panoramic radiograph [25]). Finally, *MRI* is an imaging technique that produces images of thin slices of tissue with high spatial resolution, so reducing the presence of artefacts.

Among these radiographic techniques, the most common methods for assessing bone–implant integration analysis are periapical and panoramic radiography and *CBCT*.

It can be said, on the whole, that the information derived from radiography is mostly qualitative (with the exception of *MRI* and *CBCT*); the output is mainly related to bone tissue morphology, and it cannot provide any functional information about the mechanical properties of tissues and of the bone–implant system.

### 2.2. Vibration Methods

Vibration methods are based on the behavior of mass-spring systems subjected to a transient, impulsive force or to a stationary, oscillating force.

#### 2.2.1. Theoretical Background

**Figure 1:**
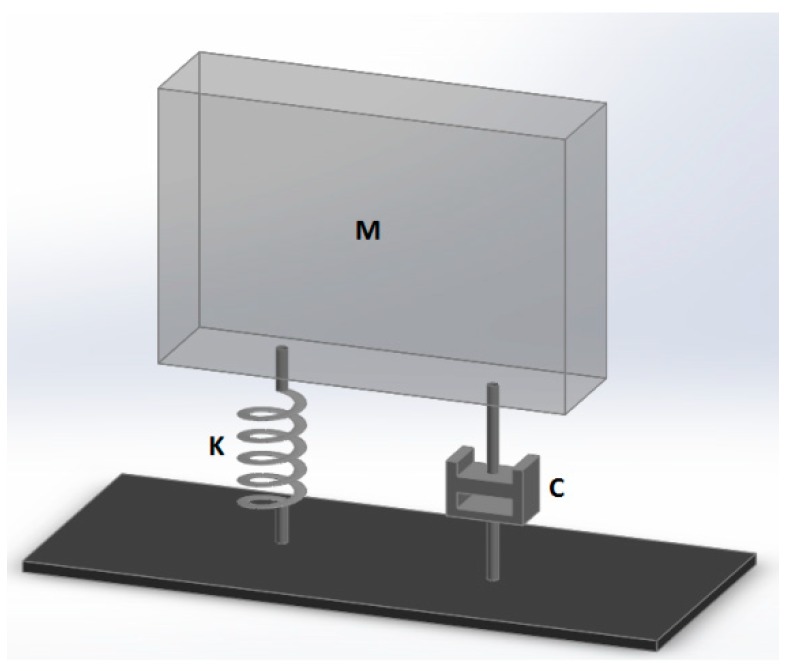
General one-degree-of-freedom system: mass *M*, spring with *K* stiffness and damping element *C*.

Given the simplified model illustrated in Figure 1, and supposing it is impacted by a general impulse ‘*I*’, the system begins vibrating, according to well-known vibration theory equations [30]:

(1) $$x\left(t\right)=\frac{I}{M\cdot 2\pi f_{d}}e^{-\zeta \cdot 2\pi f_{n}\cdot t}\sin\left(2\pi f_{d}\cdot t\right)$$

where:

- *M* is the suspended mass (it can be approximated to about 4 g for a single implant);
- *K* is the spring stiffness, whose value can be approximated between 100,000–200,000 N/m, according to [31];
- ζ is the relative damping, calculated from the damping constant *C* as *C*/[2(*KM*)^1/2^]; its value can be approximated as 0.25, according to [32];
- *f_n_* is the natural frequency of the undamped system;
- *f_d_* is the resonance frequency of the damped system.

The last two frequencies can be calculated as follows:

(2) $$f_{n}=\sqrt{\frac{K}{M}}$$

(3) $$f_{d}=f_{n}\cdot \sqrt{1-\zeta^{2}}$$

Given usual values of relative damping ζ (0.25, according to [32], these two frequencies are very close to one another and *f_d_* can be approximated as *f_n_*.

In relation to the following paragraph, it could be useful to also calculate the half-period length; that is:

(4) $$T_{1/2}=\frac{\pi }{\sqrt{\frac{K}{M}\cdot \left(1-\zeta^{2}\right)}}$$

The same system illustrated in Figure 1 could undergo ‘forced vibrations’ in the case that a continuous oscillating force is applied. Given a sinusoidal force with amplitude *F* and frequency *f*, the system response is a vibration having the same frequency *f*, and whose amplitude *X* can be so calculated:

(5) $$X=\frac{F}{K}\cdot \frac{1}{\sqrt{{\left[1-{\left(\frac{f}{f_{n}}\right)}^{2}\right]}^{2}+{\left(2\zeta \frac{f}{f_{n}}\right)}^{2}}}$$

Vibration tests are based on the assumption that the resonance frequency is directly related to the stiffness of the bone–implant interface, and of the surrounding bone: they act like two springs in series, therefore the softer one plays the greatest influence [33]. As a general rule, high values of resonance frequency are produced by successfully integrated implants, while low values may be signs of ongoing mobilization and/or marginal bone loss. Caution has been expressed by the European Association of Osseointegration (*EAO*), since it has been realized that Resonance Frequency Analysis (*RFA*) is affected not only by bone tissue characteristics, but also by the effective implant length, diameter, and surface characteristics. This is the reason why no established normative base on RFA is available yet, and the trend of resonance frequency versus time is thought to be significant, rather than its absolute value, measured at a certain time step.

It should be here emphasized how the model illustrated in Figure 1 is a conceptual model: in usual tests, the implant undergoes both rotation and translation; consequently, the mass *M*, the stiffness *K*, and damping constant *C* are ‘equivalent’ values for the dominant motion.

The physical property being inquired is different for vertical or horizontal vibrations. In the case of vertical vibrations, the shear modulus of maturing bone plays an influence as well as the ‘effective’ interface area, that is the area which participates to load transmission. In the immediate postoperative condition, the effective area is governed by friction and by interface pressure (‘press-fit’) between the implant and bone. Successively, the press-fit loosens [13,34,35] and the ‘effective’ area is the area where the bone has integrated the implant. In the case of a horizontal percussion, contact forces are sufficient to produce compressive stresses between the implant and the bone: there is no need for adhesion between these two components; in this case, test results are mostly influenced by the compressive elastic modulus of the maturing bone surrounding the implant and by the implant geometry. This aspect has been carefully considered by [36] in his in vitro experiments: he concluded that the interface should undergo shear stresses in order to achieve the highest sensitivity.

#### 2.2.2. Tests and Devices Based on Transient Force Application

The so-called ‘percussion test’ is a quite common practice in dental dentistry: the implant is hit by a sort of hammer (most usually, the back end of a mouth mirror) to produce its distinctive sound. This test is based on the assumption that an integrated implant should have a more acute and defined sound, while a not-integrated implant should have a low-frequency, dull sound. According to the most refined ‘protocols’, the implant is initially percussed vertically, and in the case of a positive outcome, the abutment is connected and a horizontal percussion is performed. The output of this test is a qualitative measure of the natural frequency of the implant; it is a subjective method, relying on the clinician’s experience. This test has been demonstrated not to provide an accurate estimate, since the successful osseointegration of 15% of the interface area is enough to produce a ‘clear’ sound [37]. Besides, seemingly ‘sound’ implants have resulted in the inability to withstand ‘test’ torques (see the following paragraphs). Implant position has a relevant influence, since dental implants in the maxilla will have a greater resonance due to the proximity of the nasal cavity and maxillary sinus.

An improved version of the percussion test is given by the so-called ‘impact hammer method’; it is still based on the use of a transient force as a source of excitement, but the sound generated by the contact with the implant is processed through a Fast Fourier Transform (*FFT*) to produce a quantitative measure that is the resonance frequency. An instrument working in this way is the Dental Mobility Checker (*DMC*), by the Morita Corporation, Japan; this device has been developed to test native teeth mobility [38,39]. A small impact hammer is here used as an excitation device, while the implant vibration is acquired through a microphone and processed through a spectrum analyzer in order to obtain the peak resonance frequency. This system is able to produce a quantitative, objective measurement. The repeatability is affected by the percussion being performed manually and by contact. This implies some drawbacks [40]: the impact location is not repeatable, and if the excitation point is close to a nodal point of the structure, the output signal-to-noise ratio can be relatively low. Secondly, the hammer hit could damage the bone–implant system. Finally, the input frequency bandwidth is limited and it could not excite the high-frequency modes of the structure (according to a recent work by Zanetti et al., low-frequency modes are related to bone deformations rather than implant displacement inside the bone [33]). Unfortunately, it was not possible to find any specifications of this device, which is no longer produced by the Morita Corporation.

**Figure 2:**
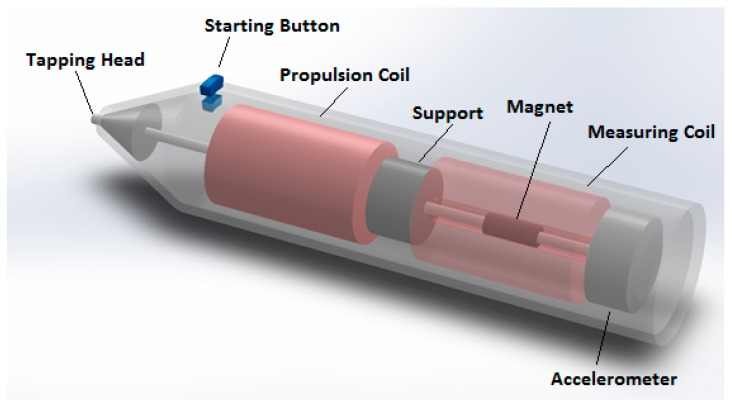
The Periotest probe.

The Periotest (Siemens, AG, Bensheim, Germany) is another device to realize a percussion test. This instrument controls the impact through an 8-g rod with an accelerometer sensor at its end (Figure 2).

The rod has a magnetic core and it is propelled at a constant velocity by means of two coils: when they are activated, the rod taps the implant abutment. The Periotest measures the time elapsed from the initial contact to the first rebound off the implant; this measure corresponds to half the period calculated in Equation (4). Time data (*T*) are converted to the “Periotest Value” (*PTV*; numerical integers) through a linear relationship (*PTV* = 50000*T* − 21.3). Data suggest that low values are produced by successfully integrated implants, while high values may be signs of ongoing mobilization and/or marginal bone loss. As well outlined by [32], the outputs of this test are mainly dependent on the stiffness of the surrounding tissues, while damping plays a secondary role, differently from assertions often found in the literature [41,42]. The clinical value of one single reading might be questionable, since factors such as bone density, implant position (upper or lower jaw), abutment length, and supracrestal implant length do play an influence. Outputs lack repeatability since they undergo significant changes as recording position and angulation of the instrument vary [42]. This is the reason why the analysis in a pattern of changes over time is advised, rather than considering absolute values [43]. The time accuracy in the establishment of time-to-rebound is equal to 0.01 ms, according to [32]; that is an accuracy of about ±10 Hz, given a resonance frequency of 1 kHz. Various authors complain that the sensitivity of this device is too low to discriminate among different osseointegration levels [43]. The Periotest was followed by the Implatest (by Q Labs, Providence, RI, USA), the main difference being that the accelerometer used to acquire implant vibrations is supported by a membrane connected to the probe’s body; this configuration allows the isolation of the accelerometer from the probe’s motion, thanks to the flexibility of the membrane. According to some authors, an integrated implant exhibits a smooth frequency response with a single main peak, while at the early stage of integration, a noisy frequency response signal is observed [44]; the same authors state that their procedure is affected by limitations coming from the use of a single uniaxial accelerometer; this could lead to imprecise measures if the implant is not completely surrounded by the bone, because the implant stiffness would change as a function of the radial percussion angle. Another similar device is the Implomates (by Bio Tech One Inc., Taipei, Taiwan), where the impact is produced by a metallic rod driven by an electromagnetic field, while the vibration is acquired by a microphone; the time response signal is analyzed in the frequency domain, in a range between 2 and 20 kHz [45,46], with 50 Hz resolution [47]. This device has been designed with the aim of minimizing the time required for the measurement and its invasiveness: it does not require any torque force application on the abutment.

**Figure 3:**
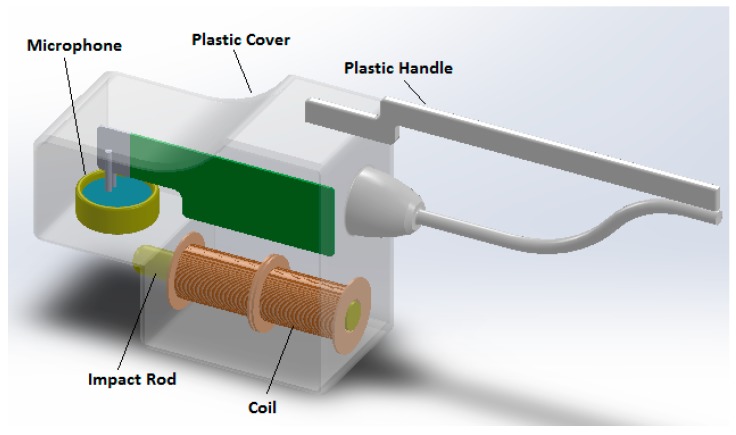
The Implomates device.

As shown in Figure 3, the device includes a minimum contact transducer and a turnable plastic handle, which can be rotated to facilitate its access in the oral cavity. The device produces approximately a 0.18 N force [47] and the resultant vibration signal is sent to a spectrum analyzer; the peak value of the vibration amplitude identifies the resonance implant’s frequency.

#### 2.2.3. Tests and Devices Based on Stationary Force Application

Vibrations of the bone–implant system can also be excited by a continuous, sinusoidal force at a fixed frequency [36] or spanning over a given frequency range; in the first case, the output is the vibration amplitude; in the second case, the output is the resonance frequency. In both cases, the results are heavily biased by load direction and position [36].

**Figure 4:**
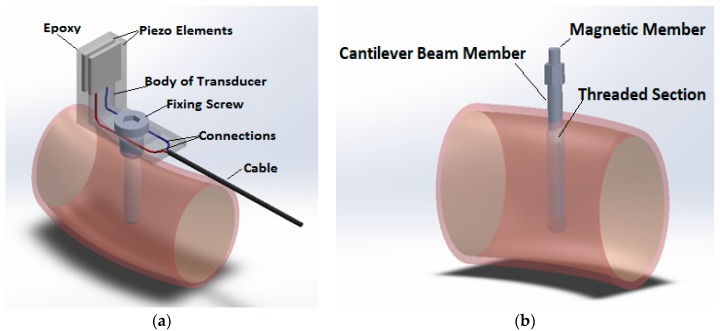
Osstell devices: (**a**) the former L-shaped sensor; (**b**) the following Osstell Mentor/IDX.

The most widely used device operating on this principle is the Osstell (Osstell AB, Columbia, Portland, OR, USA); it is similar to a recently commercialized device that is the Penguin RFA (Integration Diagnostics, Sweden). In the former version produced by the Integration Diagnostics Ltd. Company (Gothenburg, Sweden; Figure 4), this device utilized an L-shaped transducer fabricated from stainless steel or titanium [48] bearing two piezoceramic elements.

The beam was vibrated by exciting one of its elements with a sinusoidal signal of varying frequency ranging from 5 to 15 kHz, with 25 Hz steps, along two orthogonal directions; this signal was synthesized by a frequency response analyzer and a PC. The second piezoceramic element measured the response of the beam and a charge amplifier amplified the output signal. As soon as the first resonance frequency of the beam was reached, a marked increase of the output signal amplitude was recorded as well as a phase change of the signal itself. The highest frequency between those measured along two orthogonal directions is the frequency which is used to calculate the implant stability quotient (*ISQ*) through a mathematical relationship, reported in the respective patent (US 2002/0143268):

(6) ISQ = f·(k+n·L)+m·L+p

where *f* is the measured resonance frequency, *k* and *n* are calibration factors in the range −1 to +1, *m* and *p* are calibration factors in the range −100 to +100, and *L* is the abutment length. The *ISQ* scale runs from 1 to 100 units for frequencies ranging from 3000 to 8500 Hz [49]. Calibration factors are obtained from calibration blocks having different stiffness with a priori assigned *ISQ* values. When no abutments are used, *L* is 0 and the relationship (6) reduces to:

(7) ISQ = f·k+p

*k* and *p* values can be evaluated through a linear regression of *ISQ* versus *f* (*k* is the slope, and *p* is the intercept). If *k* ≠ 0, auxiliary variables are used:

A = k+n·L

B = m·L+p

These two factors are determined through linear regression of *ISQ* values versus resonance frequency. The next step is the calculation of *n* and *m* by subtraction.

The following versions of the Osstell device are based on a cylindrical peg mounted on the abutment; both forcing and output signals are taken without contacting the peg, through electromagnetic waves. Again, the recorded frequency is converted to *ISQ*. The built-in device microphone converts the acquired signal into a frequency spectrum through a *FFT* analysis; the frequencies of the first peak are recorded along two orthogonal directions. *ISQ* values are finally calculated. The respective patent (US 2014/0072929 A1) reports the following formula for *ISQ* calculation:

(8) $$ISQ=f^{2}\cdot \left(u+vL\right)+f\cdot \left(k+nL\right)+p+mL$$

where *f* is the resonance frequency; *L* is the abutment length; and *u*, *v*, *k*, *n*, *p*, and *m* are undisclosed constants, depending on the implant geometry.

However, a recent work reports a higher-order relationship [50]:

(9) $$ISQ=\left(f^{4}\cdot e\right)+\left(f^{3}\cdot d\right)+\left(f^{2}\cdot c\right)+\left(f\cdot b\right)+a$$

where *f* is the resonance frequency and coefficients *a*, *b*, *c*, *d*, and *e* are undisclosed coefficients, depending on the abutment length and implant geometry.

The constants are typical of each implant and are obtained by calibration [50], prescribing that all implants should produce similar *ISQ* values for the same boundary condition.

With reference to the last Osstell version (Figure 4b), different pegs are provided for each implant, and the respective constants are obtained by calibration [50]: the process makes use of a set of five blocks with different stability and with predetermined *ISQ* values (see US Patent 2014/0072929A1). Calibration parameters are determined so as to produce the same ISQ values for the same boundary conditions; that is, for the same stability levels. The clinical value of one single reading might be questionable [51]. For example, factors such as bone density, implant position (anterior or posterior/upper or lower jaw) [52], abutment length, supracrestal implant length [53], and implant length [52] and width [54] have been proven to play an influence on the absolute measurement value of resonance frequency. Rabel et al. demonstrated how *ISQ* values obtained from different implant systems were not comparable [55], and this could be the reason why more complex relationships have been set up for *ISQ* calculation. In any case, the prognostic value of this technique might be significant in prospective clinical studies: during the healing period, an increase of *ISQ* values has been reported when primary stability is low [56]; on the contrary, the same authors showed how *ISQ* values do not exhibit any significant change when primary stability is moderate. The position of the device should be kept orthogonal and at a distance of 3 mm from the abutment in order to get significant results. New formulations of *ISQ* reported above are finalized to obtain results independent from the implant geometry and abutment length; otherwise, only the pattern of *ISQ* versus time can be considered reliable [57]. Table 1 synthetizes the main findings concerning stability measurement devices based on bone–implant vibrations.

### 2.3. Static Force Measurement: Reverse Torque Test

Osseointegration can also be determined through the ability of the abutment to withstand a given torque value. This test was first proposed by [58] and was followed by [59], who identified the critical torque as being the torque leading to the destruction of the bone–implant interface. More recently, a more conservative approach has been followed: a torque threshold is preventively established and the test is finalized to assess whether the implant can withstand it without failure; the output of the test is therefore “pass or fail”. The same implant driver and wrench can be used for this aim: a torque equal to approximately 20 Ncm [60] or even more is applied [61]; the implant is expected to stay fixed and not rotate. Particular care must be taken during this type of examination, since it could produce maturing bone damage, especially in the posterior maxillary region: at the end, this can be classified as a ‘destructive test’, at least for all those cases when it fails; even when the implant is not mobilized, irreversible bone plasticization could take place [62]; however, the implant would soon reintegrate, according to some authors.

From a physical point of view, this test produces a shear stress to the interface area between the implant and the bone. This area can be approximated as a cylinder (radius: *r*, length: *l*) or as a taper (radius at its smaller base: *r*_0_, length: *l*, slope: *k*), and the shear stresses can be so calculated from the torque, *T*:

(10) $$T=\int_{0}^{2\pi }\int_{0}^{l}\tau_{cyl}\;r^{2}\;dz\;d\vartheta \Rightarrow \tau_{cyl}=\frac{T}{2\pi r^{2}l}$$

(11) $$T=\int_{0}^{2\pi }\int_{0}^{l}\tau_{tap}{\left(r_{0}+kz\right)}^{2}\;dz\;d\vartheta \Rightarrow \tau_{tap}=\frac{3T}{2\pi l\left[3{r_{0}}^{2}+3r_{0}kl+k^{2}l^{2}\right]}$$

The simplified formulas reported above explain why an absolute torque value cannot be given independently from the implant geometry. In fact, the same torque can produce very different shear strain on bone.

From a mechanical point of view, the reverse torque test can be put in relation with axial vibration tests: in both cases, the force transmission relies on friction forces, as long as osseointegration has not been reached, and on adhesion forces between the bone and the implant, successively.

A definite threshold to assess osseointegration has yet to be established [43], also considering the variability of implant shapes and sizes.

This test has been often used in the clinical practice since it does not require any additional device: the same implant driver and wrench can be employed; however, as said above, it bears some major drawbacks due to the risk of maturing bone damage. Some authors even speculate that crestal bone loss and failure of implants may be the result of this test, especially in less dense bone types [15]; in addition, according to the same authors, there is up to 25% uncertainty on the actually applied torque, there is still the lack of an objective measurement of the induced interface displacement, and there is no way to distinguish elastic deformations from permanent displacements at the bone–implant interface. Considering all these limitations, less invasive techniques are being preferred, even if specific devices need to be acquired.

### 2.4. Ultrasound

Quantitative ultrasound (*QUS*) is a new method, introduced in more recent times [63]. The principle of the measurement lies on the dependence of ultrasonic propagation within the implant on the boundary conditions that are the biomechanical properties of the bone–implant interface. While the above-described methods use a mechanical stimulus to induce micromovement in order to analyse the implant response, this methodology evaluates the bone tissue ingrowth into porous implant surfaces using a low-intensity pulsed ultrasound. Experimental tests have demonstrated that when a pulse with 1 MHz central frequency is launched in an aluminum screw-shaped waveguide embedded in an aluminum block, the transmission energy depends on the contact area, and moreover, the detected differences in this transmission are strong enough to identify different levels of attachment [64,65]. The probe transducer is securely attached to a healing abutment; it generates a 10 MHz broadband ultrasonic pulse and it is linked to a pulse receiver via a standard coaxial cable. The output radiofrequency signal is sampled at 100 MHz, and the average amplitude of the signal between 10 µs and 120 µs is used as an index of implant stability (*I*). The transducer is screwed into the implant with a controlled torque equal to 3.5 Ncm, well below the standard torque for implant insertion (20 Ncm), and therefore with no risk for the bone–implant interface. Given the nature of the pulsed ultrasound, which is a pressure wave, the *QUS* provides a safe noninvasive mechanical stimulus with no risk for the recovery process; in fact, low-intensity pulsed ultrasounds have been proven to stimulate osseointegration [66]. The clinical effectiveness of devices based on ultrasound has been tested on rabbits, where a good correlation between the *I* index and bone–implant contact (*BIC*) has been reported [67].

Table 2 reports a synthesis of main devices categories, their invasiveness, the accuracy of the respective outputs, and the most influent variables.

## 3. Clinical Validations

The prognostic value of *ISQ* measurements has been demonstrated by Glauser et al.: they examined 81 implants, with a failure rate of 11.1% at one year. The group of future failures showed a continuous decrease in implant stability: after one month, the mean *ISQ* value of 52 was statistically lower for the group of future failures than for the successful implants, which showed an *ISQ* of 68 [68]. Analogously, other authors demonstrated that there was a significant difference between successful and failed implants when the *ISQ* values were compared for individual implants at placement: 61.0 versus 55.9 for average *ISQ* [52]. Nedir et al. found an opposite result: unsuccessful implants’ behavior was similar to successful ones; only one week before failure, a sudden decline of *ISQ* values was recorded [54], and the respective values were only marginally and not statistically significantly outside the range of the *ISQ* values encountered for other implants. In addition, the *ISQ* value undergoes significant changes only in the first three months following implant placement [52]. This result is in agreement with a recent numerical work, where vibration methods were shown as being sensitive to osseointegration only at the very beginning of this process [33]. Nedir et al. demonstrate that Osstell could not serve as a reliable diagnostic means to identify a mobile implant with accuracy [54], and suggest an *ISQ* value of above 49 to apply the ‘delayed loading’ protocol and above 54 to apply the ‘immediate loading protocol’.

The Periotest has been demonstrated to provide a reliable prognostic measurement as well, having established a ‘−2’ cutoff [69]. However, when Periotest performance is compared to *ISQ* for follow-up assessment, the second technique results in providing more repeatable results [51,70].

In the following section, the correlations among various tests results will be analyzed in detail. Quantitative comparisons between results obtained by different devices are practically impossible, since they often measure different quantities and employ different scales. Even tests finalized to the measurement of resonance frequencies are not immediately comparable, since most of them include an additional mass (pegs mounted on the abutment, tapping rods, ultrasound probe, etc.). Nevertheless, in the following section, some studies are reported whose aim was comparing the predictive capability of each technique.

Mundim et al. [71] studied the correlation between *ISQ* values (measured through the Osstell device) and computerized texture analysis of radiographs; they found that texture attributes were significantly associated with the implant stability measures (*ISQ*) and suggest that periapical radiographs might be a reliable quantitative method for the prediction of implant stability. However, no clinical validation of any of these two methods was provided in this article. Analogously, Vayron et al. [72] found a significant correlation between *ISQ* measured by the Osstell device and marginal bone loss, as well as Miyamoto et al., who gave strong evidence of a linear relationship between *ISQ* values and cortical bone thickness [73]. Finally, Farré-Pagès et al. reported a statistically significant relationship between different bone qualities, according to Lekholm and Zarb’s classification [74] and *ISQ* values [75].

Oh et al. did not find any significant difference between *PTV* and *ISQ* and stated that both clinical measurements well correlated to the new peri-implant bone formation rate [76]; *PTV* and *ISQ* are ‘moderately correlated’, according to [77]; however, they led to partially opposite conclusions; the reason was, according to the authors, a lower accuracy of *PTV*, since a much better correlation was found in laboratory tests [69].

Implomates results were highly correlated with *ISQ* values, while producing more repeatable results [47].

Reverse torque values (that is, a reverse torque test conducted up to interface failure) proved to correlate poorly with *ISQ* values, at least with reference to primary stability [78]. This finding was confirmed also with reference to secondary stability [79]: the correlation between *ISQ* and reverse torque values was good only on subgroups of data measurements, obtained at the same time from the implant.

A recent study [80] compared the results obtained from the *QUS* method and *ISQ* values at different healing times. The ultrasound technique proved to provide a higher sensitivity to the osseointegration process: the error in the estimation of the healing time was found to be around ten times lower for *QUS* compared to *ISQ*. The authors explain this result considering that *ISQ* is related to the vibration of the whole bone–implant system, while the *QUS* response was related to bone properties on a confined volume that was about 30 µm around the implant [80]. Another study compared the results obtained with these two technologies in dental implants placed in bone-mimicking phantoms: again, *QUS* resulted to be a more accurate tool, with higher sensitivity, especially with reference to the final drill diameter [72]. Nonetheless, the reliability of the *QUS* technique still needs to be evaluated in clinical studies.

With reference to the histological analysis, it has been previously outlined how its use is limited to experimental in vivo tests, while it cannot be used in the clinical practice due to its invasiveness. Nonetheless it is interesting to report correlations between this exam and other techniques in order to provide a deeper understanding of the respective performances. Various authors have reported a poor correlation between the Periotest and histological data [81,82], since the first one is less sensitive and it can detect only large differences in bone–implant contact. Seemingly, the correlation between *ISQ* and histologic analysis is also not so good [83]; the reason may be that not only the bone–implant contact area, but also other factors such as bone density (and the respective elastic modulus) do play an influence on *ISQ* results [83].

## 4. Discussion and Conclusions

As a general rule, devices not requiring an additional element in contact with the abutment are considered to be safer: the Periotest, *DMC*, and Implomates belong to this category, while the Osstell requires screwing the magnetic peg on the top of the abutment with 10 Ncm torque, and this might affect the bone–implant interface at the early healing stage [47]. On the other hand, no-contact device results are hampered by a lack of repeatability, since small deviations in the location of the impact point result in significant variations of results [84].

Devices working with loads orthogonal to the implant axis are mostly sensitive to ‘free implant length’; that is, the distance between the implant top and the cortical crest [43,48]. Secondarily, they are also sensitive to the normal elastic modulus of maturing bone tissue.

Devices working with loads parallel to the implant axis or torque moments are mostly sensitive to the actual contact area between the bone and the implant and to the shear elastic modulus of maturing bone. The ‘actual contact area’ is governed by press-fit at low osseointegration levels and by the mechanical interlocking between the bone and the implant (that is, secondary stability) at higher osseointegration levels.

The above-reported observations suggest that these measurements are somehow complementary and could be used in conjunction; and strictly speaking, only the reverse torque test, axial vibration, and ultrasound are dependent on interface properties [68,73,85].

The actual mechanical system made of the bone and implant does not have one single mode of vibration, and this is the reason why all devices working on *RFA* specify that the ‘first’ resonance frequency should be used as a reference. However, the ‘lowest’ resonance frequency does not represent the same mode of vibration at different stages of the healing process: depending on osseointegration level, it might represent an implant displacement inside the bone (a rotation, a vertical movement, or a torsion, in the case of transversal loads, axial loads, or torque moment, respectively) or a mode of vibration of the whole mandibular/maxillar bone [35]. These last modes are influenced by external boundaries, and as such, might lack repeatability. It would be reasonable to make an effort to follow the same mode of vibration throughout the whole osseointegration process, and this would require recording different orders of resonance frequencies in spite of the ‘first’ resonance frequency.

In addition, the bone–implant system exhibits a highly nonlinear behavior; therefore, the excited modes of vibration can be different according to the displacement amplitude generated by the applied loads. The displacement amplitude is not consistent among various tests, due to multiple reasons. First of all, not all devices are able to control the applied load for different distances between the respective device and the abutment. Secondly, given different degrees of osseointegration, even the exact same loads could produce very different displacements. Working at the ‘same loads’ or at ‘same displacements’ could seem equivalent, but is actually very different. In fact, the same applied loads produce the same ‘apparent stresses’ in bone that are stresses produced in an ‘equivalent’ homogenous material (without voids). However, the ‘true stresses’ might be very different, especially with reference to the maturing bone due to a variable bone density.

**Figure 5:**
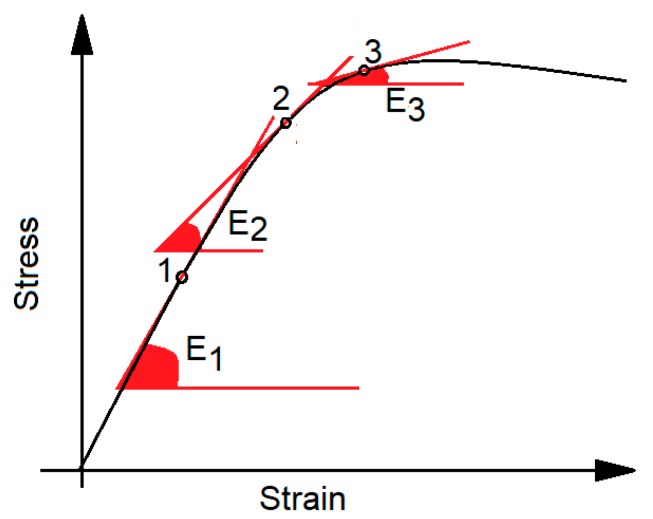
Nonlinear stress/strain curve: working at different strain (red dots ‘*1’*, ‘*2’*, ‘*3’*) implies working with different elastic moduli *E*_1_, *E*_2_, *E*_3_ that are the slope of the respective tangent lines.

Working at different ‘true stress’ values implies working on different points of the true stress/strain curve, with different tangent slopes (Figure 5) that are related to bone stiffness: in other words, even bone with the same apparent densities, and consequently similar elastic properties, would vibrate at different frequencies if different strain amplitudes are considered. Analogous considerations have led to the recommendation of using failure criteria based on strain, rather than failure criteria based on the apparent stress [86]: when strain or ‘true stress’ are used, trabecular and cortical tissues have no more apparently different properties [87,88].

As conclusive remarks, the best characterization of interface properties would require stressing the interface with both normal and shear stresses. Further efforts should be directed towards improving the repeatability of testing conditions, providing a consistency of strain amplitudes. Methods based on modal analysis should not be confined to the analysis of the ‘first’ peak. Methods based on ultrasound transmission are promising, but their clinical performance deserves to be examined in detail; to that aim, an index of stability needs to be established as well as its threshold value to ‘approve’ the implant and to allow loading. Whereas the use of the *ISQ* is nowadays well-established, a generic ‘ultrasound index’ (in ‘arbitrary units’) has been provided at the moment, but its range of variation in clinical applications is not known, and extensive tests on different implants still need to be performed.

One single value (such as the *ISQ* or *PTV*) is certainly unsuitable to give a detailed description of the whole bone–implant system, since it is the result of many factors such as cortical bone thickness, trabecular bone properties, bone–implant contact area, and so on. From this point of view, X-ray analyses are more informative, but they fail in giving biomechanical information; in addition, being able to summarize bone–implant behavior with one index is highly convenient for a fast and objective routine clinical assessment.

## Figures and Tables

**Table 1:** Main devices working on bone–implant vibrations.

Device	Input Stimulus	Measured Output	Output Sensor	Critical Issues/Disadvantages	Major Advantages
Periotest	Rod propelled by an electromagnetic driver	Contact duration	Instrumented tip	Impact location/device angulation Too-low sensitivity	Minimum invasiveness
Dental Mobility Checker	Hammer	First resonance frequenc	Microphone + RFA *	Impact location/device angulation Double tapping	Minimum invasiveness
Implatest	Rod propelled by an electromagnetic driver	Frequency response curve (smooth/noisy)	‘Floating’ accelerometer + RFA *	Impact location/device angulation Qualitative output	Minimum invasiveness
Implomates	Rod propelled by an electromagnetic driver	First resonance frequency	Microphone + RFA *	Impact location/device angulation	Minimum invasiveness
Osstell	L-truss + piezoceramic actuator	First resonance frequency	accelerometer + RFA *	Light invasiveness (the beam is screwed)	Good repeatability
Osstell Mentor/Osstell IDX	Magnetic peg + contactless actuator	First resonance frequency	Magnetic sensor + RFA *	Light invasiveness (the peg is screwed)	Good repeatability

* Resonance Frequency Analysis.

**Table 2:** Synthetic view of different devices for the assessment of implant stability.

	Invasiveness	Accuracy	Relevant Physical Quantities
**Method**	**Yes/No**	**Qualitative/Quantitative**	
X-ray	Yes	Qualitative	Bone histology and morphology
Percussion test	No	Qualitative	Bone elastic properties, implant geometry. Interface properties: + press-fit and friction/osseointegration in vertical percussion tests
Stationary vibrations	No	Quantitative (numerical)	Bone elastic properties, implant geometry, interface properties: + press-fit and friction/osseointegration in vertical percussion tests
Reverse torque	Yes	Quantitative (pass/fail)	Interface properties: + press-fit and friction/osseointegration
Ultrasound	No	Quantitative (numerical)	Surrounding bone properties
